# Contributions to the Chemistry and Histology of the Urine in the Insane

**Published:** 1856-07-01

**Authors:** W. Lauder Lindsay


### CONTRIBUTIONS TO THE CHEMISTRY AND HISTOLOGY OF THE URINE IN THE INSANE.

BY W. LAUDER LINDSAY, M.D., PERTH.

THE researches, of which the following notes contain the results, were originally undertaken with a view to study the pathology of sugar in the urine of the insane; but they soon merged into the wider general subject of the whole chemistry and histology—or, in other words, the pathology—of the urine in insanity. I have been induced to give them publicity only as a humble contribution to a subject which seems to me to have been unaccountably neglected by psychologists, believing that it is only by the labours, however apparently meagre and insignificant, of individual observers in different fields that reliable data can be accumulated whereon to base scientifically accurate conclusions. The investigation of the pathology of insanity by the microscope and test-tube will tend more and more to place mental diseases in the same category with more familiar nervous affections, and will eventually explode the crude theories as to the nature of insanity which even yet hold dominion over the public, if not professional, mind. There is a great discrepancy between observers as to the condition of the urine in insanity, and especially as to the relation of certain morbid states to particular types or phases of mental disease. These discrepancies have partly depended upon, and have partly given rise to, error; facts and theories have been too indiscriminately associated and confused. The subject, therefore, demands, and doubtless will repay, re-investigation. Much misapprehension has arisen from the overweening tendency to regard insanity as a special disease, having a special pathology and etiology, and demanding a special treatment. Fortunately this doctrine is, in many quarters, now exploded; and we are beginning to apply the same means of investigation to the study of mental, as of ordinary bodily diseases, viz., rigid scientific analysis.

The afternoted results are founded on an examination of the urine in nearly 80 insane patients—all males—labouring under almost every form of mental derangement. Dementia was the form of insanity in 45·45 per cent. of the cases examined: chronic, recurrent, or paroxysmal mania in 18·18; monomania of fear, suspicion, vanity, &c., in 22·07; melancholia in 6·49; and acute mania, general paralysis, and dipsomania in equal proportions, viz., 2·59 per cent. each. The majority were, therefore, cases of dementia, and other chronic, incurable forms of insanity; there were very few recent cases, no epileptics, and, at the period when the urines were examined, no patient laboured under maniacal excitement. The patients were in comparatively good physical health; none were confined to bed; and none laboured under specific physical diseases which could in any way interfere with the results obtained. The freedom from paroxysms, or conditions of excitement or violence, and the absence of physical disease, it is important to bear in mind, as throwing a light upon the results; for it seems probable that, hitherto, many conditions of the urine have been supposed to depend on pathological states of the brain, which have been in reality either due to increased metamorphosis of tissue from physical exhaustion, as during or after the incessant restlessness and sleeplessness of acute mania, or to functional or organic disease in other organs, as from dyspepsia, phthisis, &c. The majority of the patients were quiet and well-behaved; the mental phenomena exhibited by them were at the same time most varied. Their ages ranged from 16 to 75—the average being 43·05. All the urines examined were those passed in the morning before rising, or during the night—the *urina sanguinis*. In considering the variety of conditions which the urine of the insane presents to us, we must remember the great variability of healthy urine from the simplest causes, such as alterations in temperature or the nature of the diet, the liability to the production of morbid states by interruption to the functions of the skin and intestines, and the lower torpid vitality which so constantly accompanies or characterises chronic forms of insanity. Before giving general results, I shall review, somewhat in detail, the phenomena observed.

1. *Specific gravity.*—In 38·57 per cent. of the cases examined, the specific gravity of the urine was between 1020 and 1025; in 28·57 per cent. between 1025 and 1030; in 17·14 between 1010 and 1015; in 10·00 between 1015 and 1020; and in 5·71 between 1030 and 1040. In the majority of cases, therefore, the specific gravity was that of healthy urine. The highest specific gravity was 1038; it depended on the presence of a considerable quantity of viscid mucus, probably in the form of sputa. The presence of buccal, bronchial, and laryngeal mucus was undoubtedly also the cause of the high specific gravity in other cases. The lowest specific gravity was 1012, the urine being at the same time pale and watery-like. It occurred in three cases; two of acute mania—one, a boy of 16, the other a lad of 22—and in a case of chronic dementia, a man of 57. In all three cases the urine was acid and phosphatic; in one of the cases uric acid, and in another epithelium, were also present.

2. *Colour and smell.*—In 46·75 per cent. of the cases, the urine was noted as clear; in 16·80 turbid; in 44·15 it possessed a light amber colour; in 10·38 it was of a dark amber, or brownish red, colour; and in 31·03 it was pale and watery-like. From the proneness to enter into a state of decomposition—probably from the general presence of a certain amount of mucus—a considerable proportion of the urine speedily became ammoniacal, some very rapidly and strongly so. The formation and evolution of carbonate of ammonia in these cases were generally accompanied by the deposition of prismatic phosphates. In a number of cases there was no appreciable odour, or it was faintly diabetic; in the remainder it was ordinarily urinous.

3. *Reaction.*—In the greater number of cases, the urine was acid to test-paper. In 14·28 per cent. however, it was neutral; and in 10·38, alkaline. In the latter cases, however, it is probable that the urine was in various stages of decomposition, a considerable interval having elapsed between its evacuation and examination. The alkaline specimens were all ammoniacal and phosphatic. There is every reason to believe that all the urines would have been found acid, had they been examined at the moment of being voided. While urine may be alkaline from the presence of fixed alkalies, it is more generally so from an ammoniacal condition which is usually the result of decomposition. Indeed, it would appear from recent researches that urine, as secreted by the kidneys, is always and normally acid, but that it may be made alkaline by diet and medicines, and by disease of the urinary passages and other causes of decomposition.*

4. *Sediments.*—In the majority of cases there was no appreciable sediment. In 23·37 per cent., there was a mucous sediment, generally small in amount; in 10·38 the sediment was granular and whitish, consisting chiefly of phosphates; in 7·79 it was muco-granular, containing frequently phosphates, generally with one or other of epithelium, mucous corpuscles, uric acid, and oxalates. In 9·09 mucus was present in the form of minute floating flocculi. In a few cases the sediment consisted chiefly of epithelium and mucus; in one, of uric acid; in none, of the common urate of ammonia.

5. *Pigments.*—In 10·38 per cent. of the urines examined, a marked pink or

* Dr. Rees on "Alkaline conditions of the Urine," in "Guy's Hospital Reports," 1855.

reddish colour was developed on the addition of nitric acid, subsequent to the application of heat, sometimes in the cold. In 5·19 per cent. *blue pigment* was found in the sediment attached to cotton tubes, hairs, &c. This blue colouring matter I have observed more abundantly in other urines, particularly in that of cholera, where I have met with it both in the sediment, and as a copious deep Prussian-blue froth or scum, or simple concentration by evaporation, or on the addition of nitric acid subsequently thereto.* Chemists have not yet accurately determined the nature of this pigment. By some it is supposed to correspond with the indigo blue and cyanurin described by Haller: by others its development is thought to depend on, or to be connected with, the therapeutic action of benzoic acid in some cases.† In connexion with this subject, it is interesting to note the discovery in the urine, by Dr. Hassall, of indigo. The presence in urine of the latter substance, or of a pigment similar thereto, has lately been investigated by Sieherer, whose results, however, do not appear to be decisive as to its nature.‡

6. *Urea.*—In 53·24 per cent. of the cases, urea was easily and abundantly detected. The nitrate of urea produced, on the addition of nitric acid to the concentrated urine rapidly forming, a solid mass generally of a tawny colour, composed of an aggregated mass of tables or scales. In 36·36 per cent. the formation of the nitrate was more gradual or slow; and it occurred in the form either of a loose spongy mass, composed of an aggregation of delicate scales or plumose crystals, in that of a thin pellicle, or of floating stellæ, composed of masses of needle-shaped or plumose crystals. In the latter case the masses of nitrate frequently had a silky lustre, and fibrous radiating texture. In 11·68 per cent. the nitrate was found almost instantaneously on the addition of nitric acid, showing the presence of urea in large quantity. Of 9 patients in whom this occurred, 6 were cases of chronic dementia, 2 were recent religious melancholia, and 1 was a monomaniac [of suspicion]. The ages of the patients varied from 27 to 54, the average being 41·66. Dr. Gilchrist, of the Montrose Asylum, has brought under my notice a case in which the nitrate of urea was formed at once and in abundance on the addition to the urine cold and as excreted. I have never met with it in such abundance. When we consider that urea may be regarded, along with sulphuric acid, as an index to the amount and rapidity of the process of metamorphosis of the albuminous tissues of our bodies, and that it may be held to represent two-thirds of the whole nitrogen excreted, we must look upon the presence of such apparent excess of urea in the urine as a pathological state of some import. We know that increased activity of the respiratory function—of the oxidative process, augments the production and discharge of urea and sulphuric acid. In 10·38 per cent. it is noted that no urea was detected. This arose from the urine being in a state of decomposition, the urea having been converted into carbonate of ammonia, the elements of which were easily to be found. In these cases the urine was generally ammoniacal, fœtid, and phosphatic. In 18·18 per cent. there was marked effervescence on the addition of nitric acid to the concentrated urine; and in a like number of cases a pinkish or reddish tint was developed on adding the acid. In some cases the urea was easily detected after an interval of one or two days: more generally, however, it underwent decomposition within a short period after the urine was voided.

7. *Uric acid.*—In 16·88 per cent. of the whole cases examined, uric acid in some of its ordinary crystalline forms occurred, either in the sediment, scum,

* "The Development of a blue colouring Matter in the Urine of Cholera," "Med. Times and Gazette," May 12, 1855; or in "Histology of the Cholera Evacuations in man and the lower animals." "Edin. Med. Journal," March, 1856.

† "Pharmaceutical Journal," July, 1855.

‡ "Annalen der Chemie und Pharmacie," April, 1854.

or as a coating of the sides of the urine jar, or in all these situations. In 12·98 per cent. of the urines in which uric acid occurred, it was met with in the form of lozenges, rhombs, or conglomerate crystalline masses of a yellow tint. In 2 cases only did it occur as minute colourless lozenges and needles and in one only as small rosette-shaped crystals. It was unassociated with any other urine-sediment in 66·66 per cent. of these cases; in 16·66 it occurred along with epithelium scales and mucus corpuscles; in 8·33 per cent. phosphates, and in a like number of cases, urates occurred in the same sediment. In 83·32 per cent. of the cases in which uric acid was found in the urine, therefore, it may be said to have been the only crystalline deposit. Of 12 patients in whose urine it was detected, 8 laboured under chronic dementia, 2 under monomania, and 2 under melancholia. Their ages varied from 26 to 67, the average being 45·33. Contrasted chemically with urea, uric acid is now generally considered a transition product, the result of an inferior degree of oxidation. Hence, the formation in and discharge from the body of uric acid implies some interference with or imperfection in the respiratory function, or some abnormal excess of food or effete tissues destined ultimately to be converted into urea. Uric acid and urea differ in the laws of their metamorphosis. The former varies with the activity of the respiratory or oxidative function; it diminishes in the urine in proportion as respiration becomes more rapid and free; while the latter, under the same circumstances, becomes increased in amount.

8. *Urates.*—In only 3 cases did the common *urate of ammonia* occur, and then but to a small extent. One patient was a case of chronic mania, æt. 42: the urine was acid, of specific gravity 1032, and was turbid with urates: the sediment consisted mainly of amorphous urates, with a few epithelium scales and crystals of uric acid. Another was a case of chronic dementia, æt. 58, in which urate of ammonia was associated with the urate of soda. The third was likewise chronic dementia, æt. 40, in which urates were associated with prismatic phosphates. In the latter case they occurred in the globular form. It is matter of surprise that the urate of ammonia should have been met with so rarely, seeing that it is a frequent deposit in the healthy urine from slight changes in temperature, exercise, or diet. In one case of chronic dementia, æt. 58, *urate of soda* occurred in the scum and sediment, in the form of large, yellowish, globular masses, pierced or covered by crystalline spiculæ. The urine was neutral; specific gravity 1032; and the sediment contained, in addition, urate of ammonia and phosphates, in the form both of the ordinary prisms and enormous plumose crystalline masses.

9. *Phosphates.*—The urine was found to be phosphatic on testing by heat and nitric acid, or triple phosphates were found in the scum or sediment, in 42·85 per cent. of the whole cases examined. Of 33 patients whose urine was thus phosphatic, 15 laboured under chronic dementia, 7 under chronic mania, 3 under melancholia, 3 under monomania, 2 under acute mania, 2 under general paralysis, and 1 under dipsomania. The ages of the patients varied from 16 to 75, the average being 40·54. In 24·67 per cent. of the whole urines examined, they were found phosphatic on applying the heat and acid test: in most of these cases prismatic phosphates were likewise met with in the sediment or scum, in some cases not. In 33·76, the phosphates occurred in the scum or sediment in the ordinary prismatic form. These prisms varied much in size: sometimes they were very large and well formed; occasionally in broken fragmentary masses; rarely as minute acicular crystals. In only one case did they occur in the form of stellæ—in a paralytic labouring under the results of dipsomania, æt. 70. The urine was 1015, alkaline, pale, turbid, and contained a copious white sediment, consisting of small prisms and stellæ of triple phosphate. In 3 cases enormous crystalline masses—some of them resembling the crystals of alum—occurred: they appeared also to be phosphates in an unusual crystalline form. All were cases of chronic dementia, the ages being respectively 58, 35, and 28. In one case they were associated with ordinary prisms of the triple phosphate and with the urates of soda and ammonia: the urine was neutral, and 1032. In the second the urine was 1027, acid, and contained uric acid; and in the third the urine was alkaline, 1022, and contained a copious white sediment, consisting chiefly of common prisms. The addition of reagents, in the course of testing for phosphoric acid, magnesia, &c., developed necessarily a variety in the crystalline form of the phosphates precipitated. *Aqua ammoniæ* threw down a white precipitate, consisting generally of rosette-shaped stellæ, whose radii were frequently plumose. The stellar differ from the prismatic crystals, in containing a larger amount of phosphoric acid, the former being bibasic, the latter neutral. In the latter form, however, the phosphates usually occur when they are precipitated in consequence of the gradual development of ammonia in the urine. *Hydro-sulphate of ammonia*—probably in virtue of its alkali—also produced a copious white precipitate, likewise consisting of stellæ, associated with small squarish prisms. The limbs of the stellæ were generally granular rather than plumose, and each of their extremities was sometimes tipped with a prism, giving them a peculiar character. If the reagent was preceded by muriate of ammonia and aqua ammoniæ, the radii were more generally plumose, and the prisms were sometimes very plentiful. *Carbonate of ammonia*, followed by *phosphate of soda*, produced a white precipitate, consisting generally of beautiful crosslets or crosslet-prisms, sometimes large and plumose, at other times small and plain. The crosslets were sometimes associated with minute simple stellæ, small square prisms, acicular crystals, with globular or amorphous wreaths in small quantity, and with amorphous phosphate of lime. Similar crosslets occurred, in one or two cases, in the natural urine, on standing, but in very small quantity. The precipitate thrown down by *sulphate of magnesia* followed by muriate of ammonia and aqua ammoniæ, consisted chiefly of stellæ, some of them small and simple, their radii acicular, others larger, with plumose or moniliform limbs: associated with these were a few delicate plumes, crosslet and simple prisms. In 6·49 of the whole urines examined there was a phosphatic scum. In only one case was this distinctly and beautifully iridescent and sparkling with large prisms, which also copiously coated the sides of the urine jar. The case was chronic mania, æt. 40: the urine was neutral, pale, and 1028, and the phosphates were associated in the sediment with octohedral oxalates. Of the phosphatic urines, the triple phosphate was found alone in 53·12 per cent., and with epithelium and mucus corpuscles in 21·87, making in all 74·99 in which it was unassociated with other crystalline matters. In 12·18 per cent. it occurred along with oxalate of lime; in 6·25 with urates of ammonia or soda, and in 3·12 with uric acid. The most phosphatic urines were not those of the oldest men, or of the most acute or most chronic cases; they were peculiar to no age, and to no type or phase of insanity. At the same time it may be mentioned, in connexion with the researches of other observers, that in all cases of maniacal restlessness or excitement—which were few and mild—as well as in the only cases of general paralysis, the urine was phosphatic, though far from being the most phosphatic. It is also necessary to allude to the fact, that in the majority of cases, phosphates were found only after the urine had been allowed to stand or decompose, to a certain extent; in which circumstances healthy urine frequently exhibits the same phenomena. The extent of decomposition could generally be estimated by the intensity of the ammoniacal odour. Notwithstanding the number of cases in which the urine was phosphatic, in no one case did the earthy phosphates appear to be excreted in absolute excess. This absolute excess of phosphates, it appears from the paper of Dr. Rees already quoted, is comparatively rare: he met with it only in mollities ossium, scrofulous and ricketty children, and a few of the rarer forms of dyspepsia. The mere presence of a copious phosphatic sediment in alkaline or ammoniacal urine, is no index of the excretion of an increased quantity of earthy salts. It has been supposed that the phosphates in the urine bear a somewhat definite ratio to the phosphorus in the brain, and that, *pro tanto*, they may be regarded as the indicators of the "expenditure of nervous force." The accuracy of this conclusion, I think, admits of doubt, when we consider, not only that the phosphates appear in the urine in all forms and phases of insanity, but in every species of ordinary physical disease; in other words, under the most opposite circumstances. There is a great discrepancy between observers as to the data on which such conclusions have been based. The most recent advocate of this opinion is Dr. Sutherland, of St. Luke's Hospital, London, who asserts, as the result of a series of investigations and analyses, that a plus quantity of phosphates occurs in the urine during the paroxysms of acute mania; and that, during the exhaustive or subsequent stage, as well as in acute dementia, and in the dementia of the paralysis of the insane, a minus quantity is found, under which circumstances it denotes the "expenditure of nervous force."* I am not in a position, from my own experience, to disprove Dr. Sutherland's assertions; but it appears to me that he has taken too limited a view of the subject. Cabanis speaks of the brain in acute mania being highly phosphorescent; Couerbe found a plus quantity of phosphorus in the brain of acute mania, and a minus quantity in that of the idiot; and Dr. Sutherland states that a plus quantity of phosphates exists in the blood of acute mania, and a minus quantity of phosphorus in the idiotic brain. I must also demur to Dr. Burnett's doctrine, as at least not yet fully borne out by fact, that "to the phosphatic diathesis the greater number of forms and cases of mental disease owe their origin;" and that an excess of phosphates in the brain is "perhaps the true psycopathic interpretation of all mental exaltation."† Along with the triple phosphates was generally associated the amorphous granular phosphate of lime, which also occurred with the oxalate of lime and other urine-sediments.

In all the urines *phosphoric acid, lime* and *magnesia* were always easily detectable by reagents, but they varied somewhat in amount, more so apparently than the *sulphates* and *chlorides,* which were uniformly present in large quantity. Similar variations in the proportions present of the *phosphates* of *lime* and *magnesia* occur in various abnormal conditions of the system, and even in health. We know that the phosphates of lime and magnesia are daily excreted in the healthy urine, bearing to each other a definite ratio, which becomes altered in disease.‡ The same phosphates are also excreted in the fæces; and those leaving the body by this channel generally bear a ratio to those passing off in the urine. In connexion with the excretion of the earthy phosphates, it is interesting to remark that an unusual drain of them from the system is almost invariably attended with emaciation. Such at least has been found to be the case in strumous disease; for instance, in strumous children, whose urine frequently resembles that of the insane in containing uric acid, oxalates, and phosphates. Phosphatorrhœa, as the continuous and unusual drain of phosphates by the fæces and urine, might not inappropriately be called, is a symptom of importance in connexion with the subject of cell-growth: we know that phosphate of lime, for instance, is one of the essentials to cell-formation in the tissues. It has recently been suggested by a German physio-

* "Cases illustrating the Pathology of Mania and Dementia." "Medico-Chirurgical Transact.," vol. xxxviii., 1855.

† "On the Pathology of the Urine, and the relation which that fluid bears to other excretions in mental diseases." "Asylum Journal of Mental Science," Oct., 1855.

‡ Neubauer, "Researches on the quantity of Earthy Phosphates in the Urine." "Lancet," April 26, 1856.

logist,* that the variations in the excretion of phosphate of lime, which depends on fluctuations in the metamorphosis of tissue, constitute the chief, or a chief, cause of the differences in the weight of our bodies from day to day. In regard to the laws which regulate its production and excretion, phosphoric resembles uric acid; and the proportion excreted bears a ratio to the amount of nitrogen discharged from the system in the form of urea.†

10. *Oxalates.*—In 15·58 per cent. of the whole urines examined, *oxalate of lime* existed in the sediment, and always in the form of octohedres, sometimes large and well-formed, more frequently small, seldom in great quantity. Oxalate of lime existed, therefore, nearly in an equal proportion to uric acid. Of 12 patients in whom it occurred, 3 laboured under chronic dementia, 2 under chronic mania, 2 under monomania, 2 under melancholia, and 1 under general paralysis. The ages of the patients varied from 27 to 56, the average being 42·25. Of the urines in which oxalate of lime occurred, it was met with alone in 59·33 per cent., with epithelium in 25 per cent., and with phosphates in 16·66 per cent. In all the urines, the addition of *oxalate of ammonia* caused, on standing, the deposition of a white granular sediment, consisting of masses of minute octohedres of oxalate of lime, associated, generally, with more or less dark, amorphous, granular matter, probably phosphate of lime. In some cases a few small, delicate, prismatic phosphates were intermixed. It has frequently been asserted that the oxalic diathesis, or oxaluria, is essentially connected with lesions of the nervous system; and some authors have gone the length of associating them causatively with special forms of mental disease. But, as in the case of similar errors in regard to the phosphatic diathesis, it is probable a too limited view of the subject has been taken. The fact has not been ascertained, or has been ignored, that oxalates occur in the urine in almost every form and phase of mental disease, and in a great variety of ordinary physical diseases, that is, under the most opposite conditions; and that while they may, and perhaps do, in the majority of cases, result from the metamorphosis of tissue, or of the products of the decomposition of tissue, they may equally be derived directly from vegetable foods, or from the products of the destructive assimilation of nitrogenised foods. Oxalic acid in the urine is now generally supposed to result from the decomposition of uric acid, which, we have already seen, is produced by an inferior intensity of the oxidative metamorphosis of effete tissues than is necessary for the formation of urea.

11. *Epithelium* and *mucus corpuscles.*—In 31·16 per cent. of the urines examined, epithelium scales were present in greater or less quantity in the sediment. They were generally large, transparent, and of normal form and size; at other times they were variously elongated and shrivelled—sometimes fusiform or caudate, and dark or granular. Occasionally they were present in as great amount as in *Catarrhus vesicæ.* Of 8 patients, in whose urine an epithelial sediment occurred, 4 laboured under monomania, 3 under chronic mania, and one under general paralysis. The ages of the patients varied from 26 to 56, the average being 43·75. In 37·5 per cent. of the urines containing an epithelial sediment, the epithelium scales were associated with mucus corpuscles; in 25 per cent. with phosphates; in 25 per cent. also with uric acid; in 12·5 per cent., with urates, and in a like number of cases with oxalates. In 14·28 per cent. of the whole urines, *mucus corpuscles* were present in the sediment. They were generally associated with epithelium scales, but not uniformly. Sometimes they were very abundant, mingled with a stringy, viscid mucus: in these cases they were probably due to sputa, accidentally intermixed

* Dr. Beuche, of Göttingen, "Ueber die Wirkung des Nordser-Bactes, Eine Physiologisch-Chemische Untersuchung." Göttingen, 1855.

† Bidder and Schmidt, "Der Stoffwechsel, Eine Physiologisch-Chemische Untersuchung," Mitau and Leipzig. 1852.

with the urine. Like the epithelium scales, they were frequently aggregated in masses. They usually exhibited the reactions of pus under acetic acid; sometimes they were distinctly nucleated, the nucleus being eccentric, and becoming bifid or double under the action of acetic acid. They were sometimes associated with the "*large organic globules*" of some authors, which in the urines examined appeared to be occasionally mucus corpuscles of unusual size, but more generally bodies having a different origin and character. They sometimes resemble mucus corpuscles, except that they are much larger, and show no nuclei even under the influence of acetic acid. Usually they are more granular and dark, sometimes tinged with pigments, and they vary greatly in size. They were present in only 6·49 per cent. of the urines examined, associated or not with the "*small organic globules*" of some authorities on urinology, which were much more frequently met with under a great variety of circumstances. It appears to me to admit of little doubt that these bodies are morphologically altered blood-corpuscles, and that they can only acquire a pathological significance when present in large amount in the urine. They are generally minute, globular, colourless bodies, smaller than the blood corpuscles, but varying much in size and distinctness. *Blood discs*, unaltered, or so little altered as to be readily recognised as such, were present in only two cases. One of the patients was a monomaniac, æt. 22: the urine was acid, and 1028. The mucous sediment contained a number of small whitish or reddish specks, which were found to be bloody coagula, and which exhibited, under the miscroscope, normal red discs, with a few very transparent epithelium scales. The other was a case of chronic dementia, æt. 41: the urine was 1030, acid and phosphatic: the few slightly altered blood discs, as well as a quantity of mucus corpuscles, appeared to be derived from sputa which had got accidentally into the urine.

12. *Sugar.*—In 29·87 per cent. of the whole urines, sugar, in small quantity, and in 5·19 per cent., in large amount, was present, if *Tromner's test can be relied on,* which, however, I very much doubt. The results, though ambiguous and unsatisfactory, are, however, of such a nature as to justify, in future, the search for sugar in the urine of the insane. I have not had an opportunity of applying all the other sugar tests so as to arrive at definitive results. Meanwhile, however, I would strongly recommend the subject to the attention of psychologists and pathologists. One of my chief aims in the foregoing enquiry was to ascertain whether, and to what extent, sugar is excreted in the urine of the insane. This is not the place to detail the theoretical and analogical reasoning which led me to expect it. The four cases in which Tromner's test, that is, the sulphate of copper and potash test, appeared to indicate distinctly the presence of sugar in the urine, were 1,—Case of chronic dementia, æt. 35, urine clear, amber-coloured, 1027, phosphatic, with a copious white muco-granular sediment, containing uric acid. 2. Case of chronic dementia, æt. 58; urine 1032, neutral, phosphatic, containing urate of soda. 3. Chronic dementia, with paroxysmal mania, æt. 27, urine 1015, acid, clear, pale. 4. Case of general paralysis, æt. 42; urine acid, clear, amber-coloured, 1030, phosphatic, containing octahedral oxalates, epithelium scales, and compound granular bodies. In the majority of cases—in healthy urines, as regarded sugar, the addition of a few drops of solution of sulphate of copper merely caused a blue discoloration. On the further addition of a few drops of aqua potassæ, a greenish-blue muddiness or turbidity was produced, and a precipitate of the same colour thrown down. The subsequent application of heat merely increased or hurried the precipitate or precipitation. In some cases the flocculent, pale greenish-blue precipitate, on applying heat, rapidly became dark red, and subsided, the supernatant liquid being of a sherry colour; or it fell as a dark loam-coloured flocculent mass. In other cases the precipitate by aqua potassæ was changed to a light red or brownish red by heat, the supernatant liquid assuming a sherry colour: sometimes the liquid possessed this colour, while the precipitate remained pale or greenish. In a few cases the addition of sulphate of copper produced a precipitate [phosphate of copper] similar to that caused by aqua potassæ; a circumstance which, of itself, gave rise to the suspicion that sugar was present. Aqua potassæ dissolved this precipitate, forming an azure-blue solution or fluid, similar to that generally produced in healthy urine by the simple addition of sulphate of copper. With heat, the fluid behaved as above mentioned. These reactions varied even in the same case, according to the freshness of the urine, the period at which it was voided, and the nature and amount of the reagents added. This variability in the results is not, however, of itself surprising, seeing that Baudimont has lately found that, in diabetic urine, the sugar varies in amount, in the same case, at different periods of the day, being sometimes nearly altogether absent. For example, diabetic urine, voided in the morning, contained the merest trace, while that passed a few hours after dinner exhibited a considerable amount of sugar.

13. *Albumen, &c.*—In not a single case was the urine albuminous. All the urines were tested for the presence of *metallic salts*, without their detection in any case, by sulphuretted hydrogen, or hydrosulphate of ammonia. In a few cases *fatty matters* and *vegetable débris* were found; but these were evidently derived from the food and contained in sputa.

The chief results obtained, and the conclusions to which they point, may be shortly tabulated as follows:—

I. In the majority of cases the urines examined were clear, amber coloured, and acid; the specific gravity between 1020 and 1020; and the urea normal in amount: in other words, they were essentially healthy.

II. The triple phosphates formed the most frequent crystalline sediment; oxalate of lime and uric acid occurred in nearly an equal number of cases: epithelium, and perhaps also sugar, were present in a considerable proportion of cases.

III. While, in a large proportion of cases the urine was phosphatic, in no case was there evidence of an excessive excretion of earthy phosphates from the system.

IV. The phosphates occurred in every form and phase of mental disease, and did not appear to bear any definite ratio to the nervous energy or the "expenditure of nervous force."

V. No states or conditions of the urine appear to be peculiar to, or characteristic of, certain forms or phases of insanity.

VI. In a large proportion of cases in which sediments existed in the urine, these were not of a nature incompatible with physical health, or necessarily indicative of disease.

VII. Morbid conditions of the urine, when they were found, existed under the most opposite circumstances, both in regard to the mental and physical state.

VIII. The urine of the insane differs in no essential respect from that of the sane; and it undergoes variations from precisely similar causes.

IX. When peculiarities exist, they are more probably due to the physical than the mental state.

